# Study of Effects of Post-Weld Heat Treatment Time on Corrosion Behavior and Manufacturing Processes of Super Duplex Stainless SAF 2507 for Advanced Li-Ion Battery Cases

**DOI:** 10.3390/ma17164107

**Published:** 2024-08-19

**Authors:** Yoon-Seok Lee, Jinyong Park, Jung-Woo Ok, Seongjun Kim, Byung-Hyun Shin, Jang-Hee Yoon

**Affiliations:** 1 Semiconductor-Specialized University, Pusan National University, Busan 46241, Republic of Korea; yoonseok@pusan.ac.kr; 2 Busan Center, Korea Basic Science Institute, Busan 46742, Republic of Korea; jinyongp@kbsi.re.kr (J.P.); jwok@kbsi.re.kr (J.-W.O.); seongjunk@kbsi.re.kr (S.K.)

**Keywords:** super duplex stainless steel, corrosion behavior, post-weld heat treatment, laser welding, lithium-ion batteries

## Abstract

Lithium-ion batteries are superior energy storage devices that are widely utilized in various fields, from electric cars to small portable electric devices. However, their susceptibility to thermal runaway necessitates improvements in battery case materials to improve their safety. This study used electrochemical analyses, including open-circuit potential (OCP), potentiodynamic polarization, and critical pitting temperature (CPT) analyses, to investigate the corrosion resistance of super duplex stainless steel (SAF 2507) applied to battery cases in relation to post-weld heat treatment (PWHT) time. The microstructure during the manufacture, laser welding, and PWHT was analyzed using field-emission scanning electron microscopy, X-ray diffraction, and electron backscatter diffraction, and the chemical composition was analyzed using dispersive X-ray spectroscopy and electron probe micro-analysis. The PWHT increased the volume fraction of austenite from 5% to 50% over 3 min at 1200 °C; this increased the OCP from −0.21 V to +0.03 V, and increased the CPT from 56 °C to 73 °C. The PWHT effectively improved the corrosion resistance, laying the groundwork for utilizing SAF 2507 in battery case materials. But the alloy segregation and heterogeneous grain morphology after PWHT needs improvement.

## 1. Introduction

Li-ion batteries are widely used in various fields, from portable batteries to automotive batteries, owing to their excellent energy-storage capacity [1,2]. The recent increase in the demand for electric vehicles and portable electronic devices has led to a surge in the demand for Li-ion batteries, which, in turn, has caused numerous problems [3,4]. Among these, the high-temperature ignition of Li-ion batteries is a critical issue that directly affects human safety, prompting extensive research efforts to enhance safety [5,6]. For example, researchers such as Koleva have studied battery temperature systems to prevent ignition, whereas others have focused on catalysts to control battery fires [6]. Despite these efforts, there is a noticeable gap in the research on Li-ion batteries. Improving the materials used in battery applications can significantly enhance their safety.

Traditionally, Li-ion batteries have utilized plastic to achieve a light weight; however, this material was later changed to aluminum to improve safety. However, during a battery fire, temperatures can rise to 700 °C and the melting point of aluminum is 670 °C, rendering it ineffective in maintaining safety [3,5]. SAF 304 stainless steel is currently used for this purpose [7,8]. Owing to its extensive worldwide use, SAF 304 has been widely researched and applied in various fields, from household appliances to cookware [8]. However, SAF 304 is prone to issues such as martensitic transformation induced by plastic deformation, intergranular corrosion, and low high-temperature strength (170 MPa at 700 °C), necessitating careful attention to maintain safety. Research on the improvement of battery case materials is insufficient; therefore, further studies are necessary in this area.

Improving the materials used in battery cases is crucial for enhancing safety. Among the various materials used, stainless steel stands out because of its high strength and corrosion resistance. Based on their primary phases, stainless steels are classified as austenitic, ferritic, martensitic, or duplex [9,10]. Although austenitic steels exhibit excellent corrosion resistance, they face various issues such as those observed with SAF 304 (under 1 year in sea water) [7,8]. Ferritic and martensitic steels (e.g., SAF 409 and SAF 430) possess high strength (550 MPa) but suffer from low corrosion resistance (less than one year in seawater) and high-temperature strength (150 MPa at 700 °C), making them unsuitable for environments with diverse corrosive exposures [11,12]. However, there are no existing cases of using SDSS SAF 2507 for battery cases in the related research.

In contrast, duplex stainless steels such as SAF 2507 offer superior strength (780 MPa), corrosion resistance (50 years in seawater), and high-temperature strength (340 MPa at 700 °C), making them suitable for battery applications [13,14]. However, there are no reported cases of duplex stainless steel being used in Li-ion batteries. Duplex stainless steels composed of austenite and ferrite phases provide a balance between high strength, corrosion resistance, and high-temperature strength [15,16]. They are categorized based on their resistance index, which is calculated according to their Cr, Mo, and N contents, into four grades—lean (≤30), standard (30–40), super (40–50), and hyper (≥50). Super-duplex stainless steels (SDSSs) such as SAF 2507 have been extensively studied since the 1990s. Nilsson analyzed the microstructure and corrosion resistance of SDSSs after heat treatment, whereas Fande investigated the properties of SDSSs after laser welding [17]. While the existing literature has extensively explored the use of SDSSs, there are no instances of studies on laser welding SDSSs, as well as on post-weld heat treatment (PWHT) and corrosion behavior.

To apply SDSS to battery cases, comprehensive research on its microstructure and corrosion resistance after processing is required [18,19]. Welding results in a cast microstructure that differs from the base material, potentially causing various problems. Owing to the heterogeneous microstructure of SDSS after welding, PWHT is required to ensure uniformity and enhance corrosion resistance.

This study focused on the laser welding of SDSS, followed by heat treatment, to investigate its microstructure and electrochemical properties as a function of the heat treatment duration to manufacture advanced Li-ion battery cases. The microstructure was analyzed using field-emission scanning electron microscopy (FE-SEM), electron backscatter diffraction (EBSD), and X-ray diffraction (XRD). The chemical composition was analyzed using energy-dispersive X-ray spectroscopy (EDS) and electron probe microanalysis (EPMA). The electrochemical properties were assessed using the open-circuit potential (OCP) test, the potentiodynamic polarization test, and critical pitting temperature (CPT).

## 2. Experimental Method

### 2.1. Material

The material used in this study was cast in an electric furnace and its chemical composition was controlled. The primary alloy components (Table 1) were analyzed using inductively coupled plasma mass spectrometry (ICP-MS, Thermo Fisher Scientific, Waltham, MA, USA) [13,20]. The chemical composition corresponded to that of typical SAF 2507, with a pitting resistance equivalent number (PREN) of 42, classifying it as an SDSS. The PREN was calculated using the following formula [21,22]:

PREN = wt% Cr + 3.3 wt% Mo + 16 wt% N.(1)

Chromium is a fundamental element forming the passivation layer in stainless steel, with a lower potential (−0.91 V) compared to iron (−0.44 V), facilitating the formation of a Cr oxide layer [21,23,24]. Molybdenum has a larger lattice size than Fe, which accelerates the passivation layer formation. Nitrogen is an interstitial element that decreases Cr segregation and accelerates the passivation layer formation.

### 2.2. Manufacturing Process

**Figure 1:**
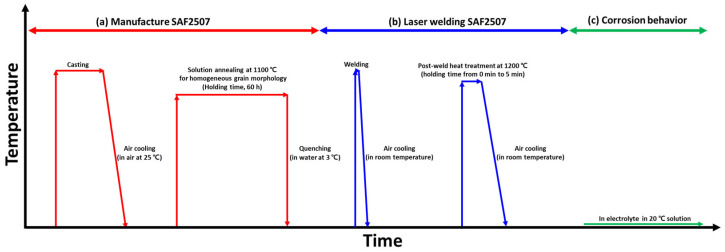
Schematic diagram for studying the corrosion behavior with post-weld heat treatment time of SAF 2507: (**a**) manufacturing process of SAF 2507, (**b**) laser welding process, and (**c**) analysis of corrosion behavior at 25 °C in 3.5, and 5.85 wt NaCl electrolyte solution.

The experimental procedure for manufacturing the SDSS SAF 2507 (size: 50 mm × 50 mm × 1 mm (thickness)), along with the welding conditions and electrochemical analysis results, are illustrated in Figure 1 [13,25]. The casting was performed at temperatures exceeding 2000 °C, followed by air cooling. After casting, the specimens were subjected to a solution heat treatment to stabilize their microstructures and chemical compositions using a field-emission scanning electron microscope (FE-SEM, SUPRA 40VP system, Zeiss, Oberkochen, Germany) and energy-dispersive X-ray spectroscopy (EDS, SUPRA 40VP system, Zeiss, Oberkochen, Germany). The solution heat treatment was applied at 1100 °C for 1 h, followed by water quenching [26,27].

### 2.3. Laser Welding

**Figure 2:**
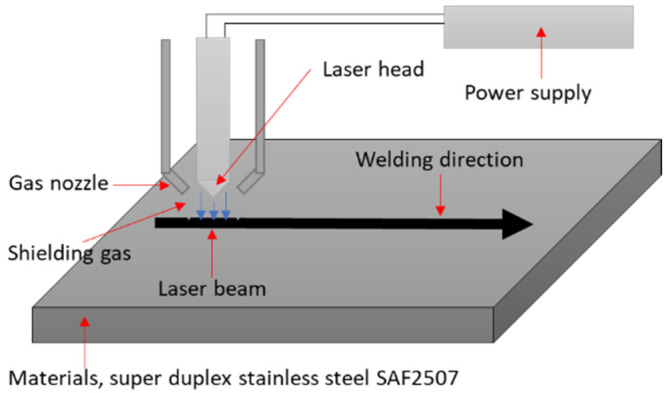
Schematic diagram of laser applied to SDSS SAF 2507.

Laser welding, which is known for its superior quality, has been employed to weld various metallic materials. A schematic of the laser welding setup is shown in Figure 2. Welding was performed at a power level of 1 kW and a speed of 1 m/min (full penetration conditions of 1 mm SDSS SAF 2507 plate). Commercial argon gas (99.9% pure) was used as the shielding gas to prevent the oxidation of the weld [28,29]. The weld zone was analyzed by examining both the surface and cross-section [30]. The state of the weld was analyzed using FE-SEM, and the surface was etched in a 10 wt% KOH electrolyte solution at 5 V for 30 s to reveal the microstructure [31,32]. The composition of the weld zone was analyzed using EDS and electron probe microanalysis (EPMA, JXA-8530F, Tokyo, Japan) on both the cross-section and surface.

### 2.4. Post-Weld Heat Treatment

Post-weld heat treatment (Figure 1c) is crucial for altering the weld microstructure and restoring corrosion resistance [29,33]. Ferritization (volume fraction over 60%) occurs at a temperature of 1200 °C but due to air cooling, the austenite grows during cooling. The heat treatment was conducted at 1200 °C for durations of 1, 3, and 5 min, followed by air cooling. The microstructures resulting from different heat treatment durations were analyzed using FE-SEM and electron backscatter diffraction (EBSD, SUPRA 40VP system, Zeiss, Oberkochen, Germany), and the composition was examined with EPMA [34,35]. The surface was etched with a 10 wt% KOH electrolyte solution at 5 V for 30 s to reveal the microstructure. The phase fractions were determined according to ASTM E1245 by averaging five measurements at a magnification of 200× [36,37,38]. To analyze the effect of microstructural differences after welding on hardness, we measured the hardness of welded specimens and PWHT specimens. The hardness was measured using a Vickers hardness tester (Mitutoyo, Kanagawa, Japan). Since the laser weld zone has a narrow weld width, the measurements were taken after moving 0.2 mm from the initial measurement position and then moving downward.

### 2.5. Corrosion Behavior

The corrosion behavior was assessed by measuring the electrochemical changes in the metal in the electrolyte solution. A potentiostat and a three-electrode cell were used for the analysis, comprising a working electrode (WE, specimens), reference electrode (RE), saturated calomel electrode (SCE), and counter electrode (CE, Pt mesh, 20 mm × 20 mm). The measurement area for each specimen was 1 cm^2^ [39,40].

The EMF series is used to determine the potential required to induce oxidation and reduction reactions in pure metals; however, the potential of the alloy was measured using the OCP. The scan rate was 0.5 s, and measurements were taken for up to 3600 s. A 3.5 wt% NaCl electrolyte solution was used, in accordance with ASTM G 61 [41,42,43].

Potentiodynamic polarization tests measured the change in the current density with the applied voltage to analyze the corrosion behavior of the specimens. The potential range was −0.6 V to +1.2 V, with a scan rate of 0.167 mV/s. A 3.5 wt% NaCl electrolyte solution was used, as specified by ASTM G 61 [43].

Stainless steels containing over 12 wt% Cr form a passivation layer consisting of Cr oxide [9,13]. However, this passivation layer can be compromised by Cl ions, leading to pitting corrosion, which adversely affects product longevity. The CPT was measured by observing the changes in the current density with temperature, which indicated the temperature at which stable pitting occurred. The pitting of surface growth increased the current density, and the CPT was determined when the current density exceeded 100 μA/cm^2^ for over 1 min. The electrolyte used for the CPT measurement contained 5.85 wt% (1 mol) NaCl, and a heating rate of 1 °C/min was used, as specified by ASTM G150-99 [44,45].

## 3. Results

### 3.1. Manufacture of SAF 2507

**Figure 3:**
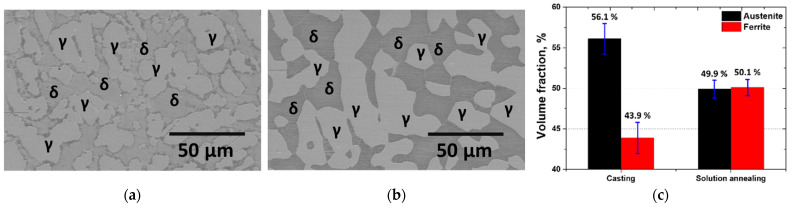
FE-SEM image and volume fraction of manufactured SDSS SAF 2507: (**a**) cast microstructure, (**b**) solution-annealed microstructure, and (**c**) volume fractions of austenite and ferrite.

The SDSS (SAF 2507) was cast in an electric arc furnace and was subjected to solution heat treatment at 1100 °C [13,46]. The microstructures and phase fractions of the manufactured SAF 2507 are shown in Figure 3. The as-cast microstructure exhibited an uneven distribution of austenite (γ). After the solution heat treatment, the austenite stabilized, resulting in changes in the phase fractions. The fraction of austenite decreased from 56% post-casting to 50% after solution heat treatment. The optimum corrosion resistance was achieved when the austenite fraction was 50%, because austenite and ferrite then had equal PRENs.

To verify the change in the PREN due to the volume fraction change after the solution heat treatment, the chemical composition was analyzed using EDS; the results are listed in Table 2. Because EDS is not suitable for the quantitative analysis of interstitial elements, the nitrogen content in the ferrite was fixed at its maximum, and the following formula was used for the calculation:

Nr = chemical composition of N Total wt% − FerriteVF × 0.05 wt%(2)

Calculating the chemical composition of nitrogen for each phase according to this formula revealed the chemical compositional changes that influenced the PREN [28,30]. The chemical composition of the primary alloy varied after heat treatment. Following the solution heat treatment, the concentrations of Cr and Mo in the austenite increased as the volume fraction of austenite decreased, whereas the concentrations of Cr and Mo in the ferrite decreased with a decrease in the volume fraction of ferrite. These changes in the chemical composition led to variations in the PREN [27]. In the as-cast condition, the high-volume fraction of austenite had a lower PREN, whereas the ferrite had a higher PREN. This PREN disparity could induce galvanic corrosion and reduce the corrosion resistance.

The solution heat treatment equalized the PRENs of the austenite and ferrite, potentially decreasing the galvanic corrosion. Consequently, the chemical composition of SAF 2507 after the solution heat treatment represented the optimal condition for maximizing the corrosion resistance, yielding a stable corrosion resistance profile [11,47]. This alignment of the PRENs for the austenite and ferrite phases ensured that the material had enhanced and balanced corrosion-resistance properties.

### 3.2. Effect of Laser Welding

**Figure 4:**
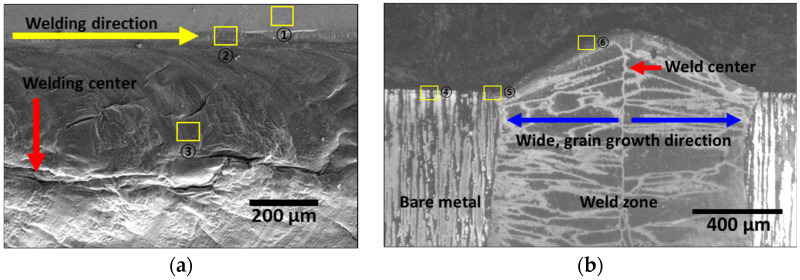
Laser-welded microstructure image of SDSS SAF 2507: (**a**) surface image and (**b**) cross-sectional image.

Laser welding, which is known to produce a superior weld quality, is an appropriate process for manufacturing batteries. Surface and cross-sectional images of the laser-welded zone are shown in Figure 4 [14,29]. The weld zone formed a bead along the welding direction (yellow line in Figure 4) with an overall width of 1200 μm. Spherical inclusions were observed on the sides of the weld zone. The weld bead height reached 160 μm along the welding direction, with a width (blue line in Figure 4) of 250 μm extending symmetrically from the center (red line in Figure 4) of the weld. Although the weld zone exhibited a narrow weld width, the heat from the welding induced the formation of inclusions on the surface. These inclusions could negatively affect the corrosion resistance [48].

To assess the corrosion resistance of the laser weld zone using the PREN, the surface and cross-section chemical compositions were analyzed; the results are listed in Table 3. The locations for the compositional analysis are marked in Figure 4 (surface: points 1–3, cross-section: points 4–6). The surface of the base material exhibited a low O concentration of 3.5 wt%, whereas the O concentration increased closer to the weld zone [14,29]. This increase in the O concentration indicated the rapid oxidation of the surface closer to the weld zone. Although the shielding gas protected the weld during welding, the residual heat led to rapid oxidation after welding. The chemical composition trend in the cross-section mirrored that of the surface, with the base material exhibiting a low O concentration of 2.1 wt%, with higher O concentrations near the center of the weld zone [49].

**Figure 5:**
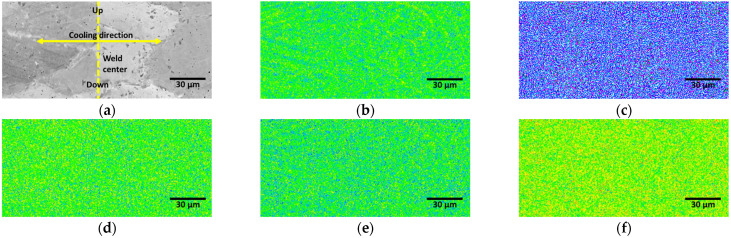
EPMA image of weld zone after laser welding of SDSS SAF 2507: (**a**) SEI image, (**b**) Ni concentration, (**c**) N concentration, (**d**) Cr concentration, (**e**) Mo concentration, and (**f**) Fe concentration.

The microstructure of the SAF 2507 weld zone, which rapidly cooled after the welding process, consisted of 95% ferrite and 5% fine austenite [50]. Figure 5 shows the concentration of major alloys. The weld zone microstructure did not exhibit any significant segregation of the primary alloying elements. Although the oxidation levels differed depending on the position within the weld zone, no internal segregation was observed.

The laser weld zone had a weld width of 1200 μm and weld bead height of 160 μm. Despite the low heat input characteristic of laser welding, the surface oxidation proceeded at a faster rate than that of the base material, resulting in a higher oxygen content in the weld zone (surface: 15.4 wt%, cross-section: 12.6 wt%). The rapid cooling rate of the weld zone led to a high ferrite fraction (95%), and no significant segregation of the primary alloying elements was observed.

### 3.3. Effect of Post-Weld Heat Treatment

**Figure 6:**
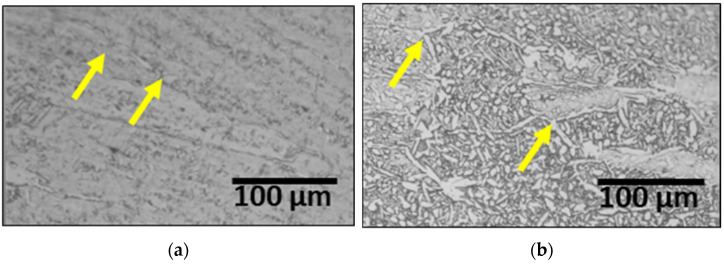

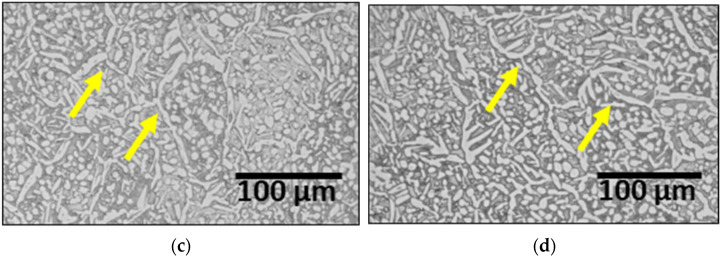
Microstructure images of laser-welded SDSS SAF 2507 after various PWHT times: (**a**) 0 min, before post-weld heat treatment, (**b**) 1 min, (**c**) 3 min, and (**d**) 5 min.

PWHT is crucial for promoting austenite growth and reducing alloy segregation, thereby restoring corrosion resistance [14,29]. After laser welding, the specimen was heat-treated at 1200 °C, followed by air cooling. The microstructural changes at different heat treatment times are illustrated in Figure 6. The austenite coarsened rapidly for 5 min, and growth along the grain boundaries was observed [13,50]. The yellow arrows indicate the initial austenite grain boundaries, with noticeable austenite growth as the heat treatment duration increased.

**Figure 7:**
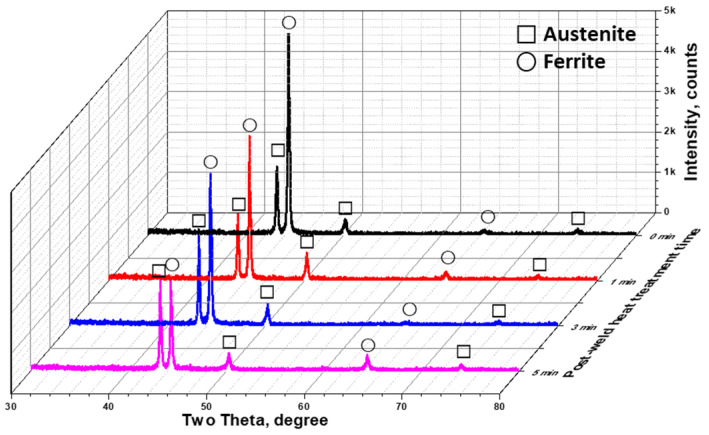
XRD pattern with post-weld heat treatment time of laser-welded SDSS SAF 2507.

The transformation between austenite and ferrite influenced the XRD patterns, as shown in Figure 7 [51,52]. Both phases exhibited a main peak in the (111) direction of growth. Before heat treatment, the intensity of the ferrite peak at 46° (111) exceeded 4400 counts, which decreased with an increase in the heat treatment duration. Conversely, the austenite peak intensity at 44° (111), which was initially at 800 counts, increased with the heat treatment. These intensity changes in the XRD patterns reflected variations in the volume fractions. The changes in the volume fractions of austenite and ferrite due to the PWHT were confirmed by both FE-SEM and XRD, and the PRENs were analyzed to determine the optimal heat treatment time that equalized the PRENs for the two phases.

**Figure 8:**
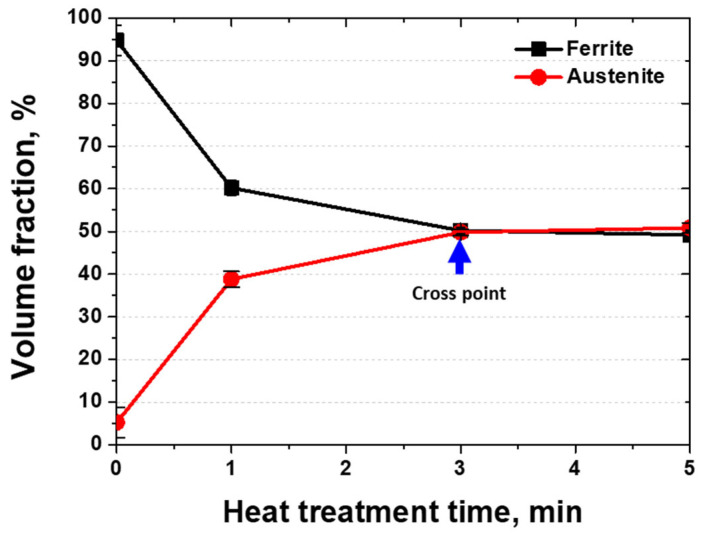
Volume fraction vs. post-weld heat treatment time curve of laser-welded SDSS SAF 2507.

The growth of austenite after the post-weld heat treatment and the corresponding volume fractions were measured, and the results are shown in Figure 8. After 1 min of heat treatment, 35% of the ferrite was transformed into austenite [13]. When the heat treatment duration reached 3 min, the volume fractions of austenite and ferrite became equal. The transformation from ferrite to austenite at 1200 °C within 3 min indicated that the phase transformation was facilitated by the heat treatment followed by air cooling. The equal volume fractions of austenite and ferrite at 3 min resulted in equal PRENs, which optimized the corrosion resistance.

Table 4 resents the chemical compositions and PREN as functions of the heat treatment time [48,53]. The chemical composition changes corresponded to variations in the volume fractions, which, in turn, influenced the PREN. The main alloying elements in the austenite exhibited a decreasing trend, whereas those in the ferrite increased. Consequently, the PREN for the austenite decreased, and that for the ferrite increased. The PREN values for the austenite and ferrite converged after 3 min of heat treatment, indicating stabilized PREN. The stabilization of the PREN at 3 min contributed to the optimization of the corrosion resistance.

### 3.4. Effect of PWHT on Corrosion Behavior

**Figure 9:**
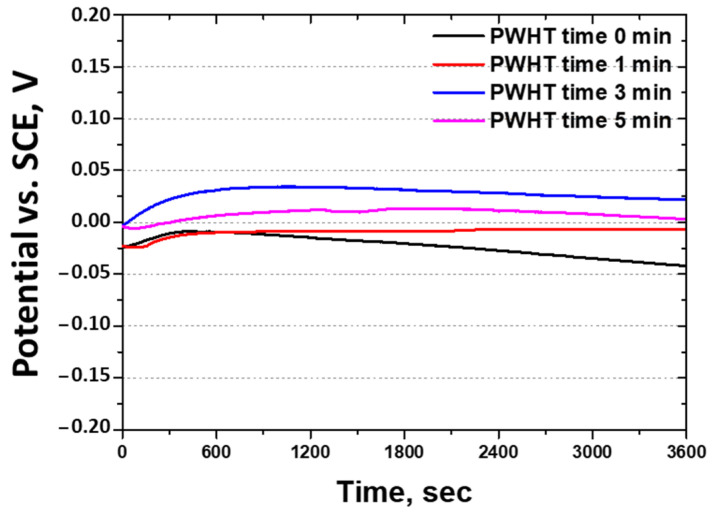
Potential (V) vs. time (s) curve: OPC curve with post-weld heat treatment time of laser-welded SDSS SAF 2507 in 3.5 wt% NaCl electrolyte solution.

The corrosion behavior as a function of the heat treatment time was analyzed using OCP, potentiodynamic polarization, and CPT tests [46,54]. Although the potentials of pure metals can be calculated, alloys require empirical measurements; hence, the OCP was utilized to determine the potential. The OCP results are shown in Figure 9 and Table 5. The potential varied with the PWHT time, with the highest potential (0.03 V) shown at 3 min, which aligned with the PREN results. The potential of SAF 2507 is influenced by the PREN, necessitating an appropriate heat treatment to minimize this potential. Therefore, the optimal heat treatment time required to decrease the reactivity of SAF 2507 was determined to be 3 min, which agreed with the PREN.

**Figure 10:**
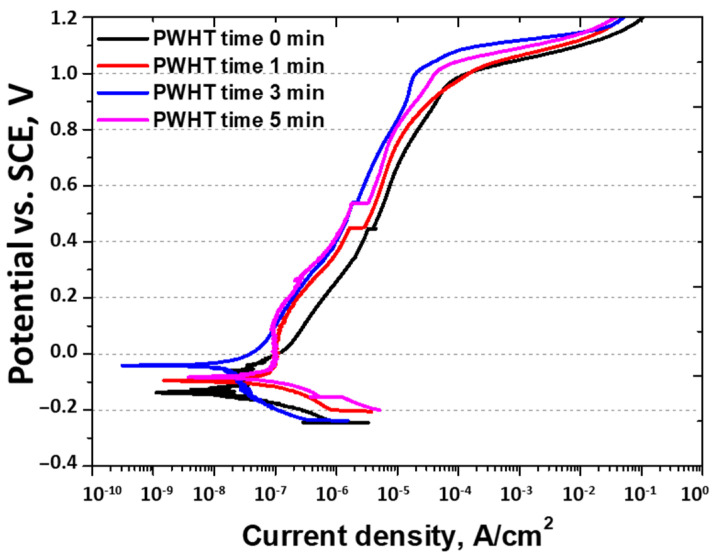
Potential (V) vs. current density (A/cm^2^) curve: potentiodynamic polarization curve with post-weld heat treatment time of laser-welded SDSS SAF 2507 in 3.5 wt% NaCl electrolyte solution.

Potentiodynamic polarization tests illustrated the change in the current density with the applied potential, showing the corrosion behavior of the specimen [31,43]. The results are shown in Figure 10 and Table 5. The potential during the activation polarization, which involved reduction and subsequent oxidation reactions, showed a trend consistent with that of the OCP results [27,39]. The current density (I_corr_) increased for up to 3 min and then decreased after 5 min, indicating the influence of the PREN on E_corr_ and I_corr_. The passivation zone showed a uniform corrosion behavior with low corrosion rates as oxidation and re-passivation occurred repeatedly. A high pitting potential (E_pit_) of 1 V was observed, indicating that the highest E_pit_ value at 3 min was influenced by the PREN. The three major values from the polarization curve indicated the best corrosion behavior at 3 min of PWHT, although the corrosion resistance was lower than that of the base material. Hence, while PWHT improves the corrosion resistance, the presence of the base material may cause galvanic corrosion owing to the lower corrosion resistance of the PWHT-treated material because of galvanic corrosion at the interface.

**Figure 11:**
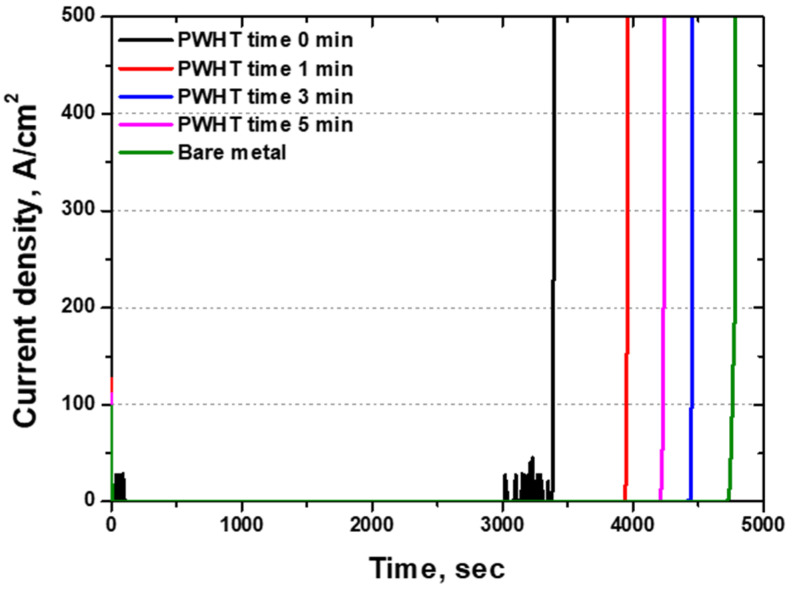
Current density (μA/cm^2^) vs. time (s): critical pitting temperature curve with post-weld heat treatment time of laser-welded SDSS SAF 2507 in 5.85 wt% NaCl electrolyte solution.

The corrosion resistance of the stainless steel was maintained by the passivation layer, and the CPT test compared the points at which this layer broke down [19,46]. The results of CPT with PWHT time are shown in Figure 11. The as-welded ferritic microstructure showed the lowest CPT at 56 °C, with uneven pitting growth observed both initially and immediately before passivation layer breakdown. After 3 min of PWHT, the CPT increased to 73 °C. This trend was consistent with the results of the other two tests. Thus, the OCP, potentiodynamic polarization, and CPT tests indicated that the highest passivation layer and the optimized corrosion resistance were achieved after 3 min of PWHT.

The electrochemical properties of the existing materials (Al 1005 and SAF 30400) were compared, and are presented in Table 6. SDSS SAF 2507 demonstrates a higher OCP, indicating it is decreased tendency of corrosion reactions than Al 1005 and SAF 30400. Its high E_pit_ and CPT values show that it is more resistant to corrosion compared to traditional materials. Using SAF 2507 for Li-ion battery cases improves the corrosion safety. Furthermore, the welds of SAF 2507 exhibit recovered corrosion resistance after PWHT, making it a suitable replacement for the existing materials.

**Figure 12:**
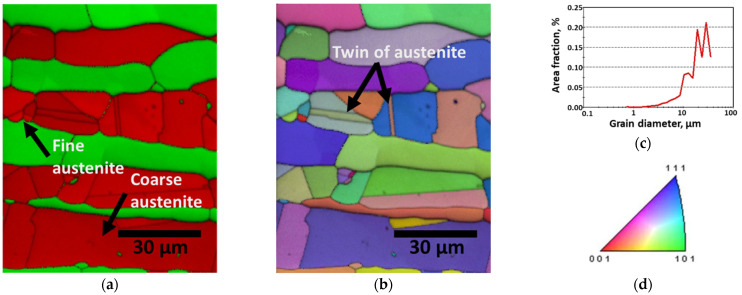
EBSD results after post-weld heat treatment of laser-welded SDSS SAF 2507 at 1200 °C for 3 min: (**a**) IPF-IQ map (red: austenite, and blue: ferrite), (**b**) phase-IQ map, (**c**) area fraction (%) vs. grain diameter (μm) curve, and (**d**) stereo-graphic triangle of IPF color map.

Despite having equal PRENs based on the chemical composition, the corrosion resistances of the solution-treated and PWHT-treated SDSS SAF 2507 differed. To understand the reasons for the decreased corrosion resistance after the PWHT, the microstructures and chemical compositions were analyzed using EBSD and EPMA. Figure 12 shows an EBSD image of the SAF 2507 after being heat treated at 1200 °C for 3 min. In Figure 12a, the austenite and ferrite are shown in red and green, respectively. The austenite growth resulted in the formation of both coarse and fine austenite grains. The coarse austenite, with diameters of 20–30 μm, contained fine ferrite grains smaller than 5 μm. Fine austenite grains smaller than 10 μm exhibited different lattice orientations, which were caused by incomplete integration during growth. Some coarse austenite grains had twins of less than 3 μm in width, which increased the dislocation density and decreased the corrosion resistance.

**Figure 13:**
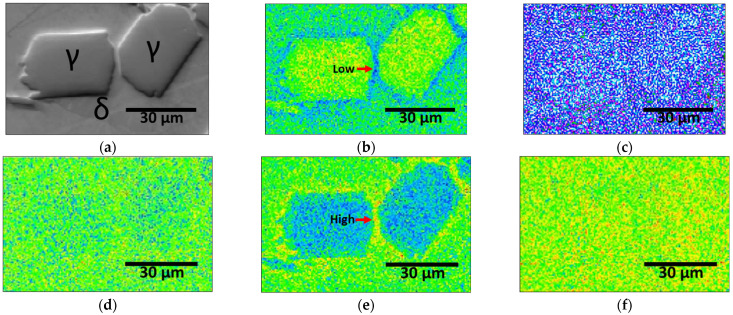
EPMA images after post-weld heat treatment of laser-welded SDSS SAF 2507 at 1200 °C for 3 min: (**a**) SEI image, (**b**) Ni concentration, (**c**) N concentration, (**d**) Cr concentration, (**e**) Mo concentration, and (**f**) Fe concentration.

The EPMA analysis results shown in Figure 13 reveal the distribution of the key elements [44,46]. Ni and Mo showed segregation at the grain boundaries, with the Ni depleted (blue) and Mo enriched (red and yellow) at the austenite boundaries. This alloy segregation affected the solubility of the Ni and Mo in the austenite, influencing the PREN and decreasing the corrosion resistance, despite an overall equal composition.

**Figure 14:**
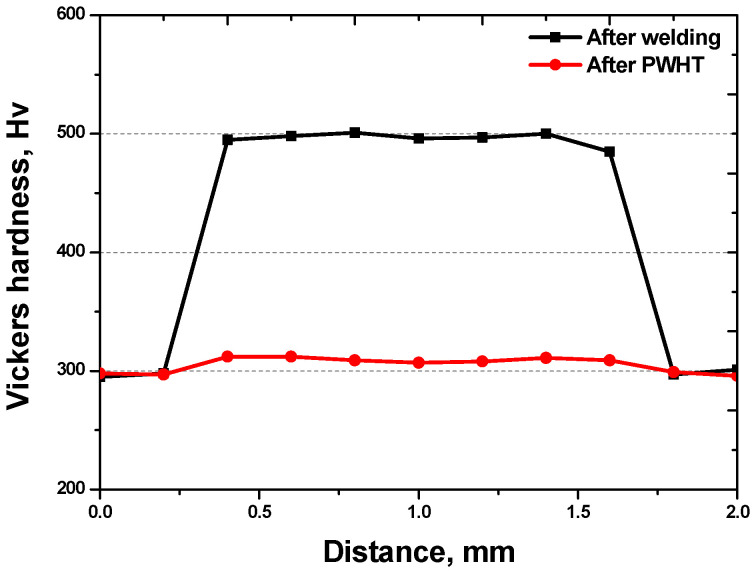
Vickers hardness of weld zone after laser welding and PWHT of super duplex stainless steel SAF 2507.

The hardness after welding significantly increased from 300 Hv to 500 Hv of SDSS SAF 2507 (Figure 14). This increase in hardness in the weld zone is attributed to ferritization. The elevated hardness decreased to 310 Hv after PWHT (Figure 14). The increase in hardness after welding is due to ferritization, as the hardness of ferrite was confirmed to be 500 Hv. In contrast, the reduction in hardness after PWHT is attributed to the growth of austenite. Although the hardness decreased after PWHT, it did not recover to the level of the base material, due to the recovery of the microstructure as a result of PWHT. However, the hardness increased because the microstructure remained fine, and alloy segregation occurred compared to the base material.

PWHT at 1200 °C produced microstructural changes and corresponding corrosion-resistance variations. Despite the restored volume fraction and PREN after the PWHT, the corrosion resistance was lower than that of the base material [19,53]. The base material exhibited superior corrosion resistance compared to the PWHT-treated SAF 2507, which showed defects in the microstructure and composition. The microstructure exhibited uneven grain sizes and fine twins, whereas the chemical composition exhibited Ni and Mo segregation. Thus, even with equalized PRENs after PWHT, the uneven microstructure and alloy segregation reduced the corrosion resistance of SAF 2507.

## 4. Conclusions

To assess the applicability of SDSS SAF 2507 for Li-ion batteries, we analyzed its microstructure and corrosion resistance after casting, solution annealing, laser welding, and PWHT. The following conclusions were drawn.

(1) The as-cast microstructure of SDSS SAF 2507 was heterogeneous, but solution heat treatment resulted in uniform austenite grains of 20–30 μm. Solution annealing at 1100 °C produced equal volume fractions and PRENs, leading to a decrease in the galvanic corrosion and improved corrosion resistance. Laser welding produced a narrow weld width of 1200 μm and a low bead height of 160 μm. However, rapid cooling induced a ferritic microstructure in the weld, with ferrite growing in the direction of the cooling (toward the base metal), comprising 95% of the weld. Despite the use of a shielding gas to prevent oxidation, the high heat during cooling resulted in a thick oxide layer on the weld surface.
(2) The PWHT increased the proportion of austenite. The austenite growth was characterized by coarse austenite along the ferrite grain boundaries and fine austenite within the ferrite grains. After 3 min of PWHT, the volume fractions of austenite and ferrite intersected at 50%, as did their PREN values. During the 3 min of PWHT at 1200 °C, the high volume fraction of ferrite (95%, metastable state) facilitated a phase transformation from ferrite and austenite.
(3) The corrosion resistance after the PWHT was measured using the OCP (−0.21 V to +0.03 V), potentiodynamic polarization tests, and CPT (56 °C to 73 °C). All the corrosion tests showed that PWHT improved the corrosion resistance, although it remained lower than that of solution-heat-treated SAF 2507. The decreased corrosion resistance was attributed to grain refinement, heterogeneity, increased twinning, and the segregation of Mo and Ni. Consequently, although the PWHT restored the corrosion resistance of SAF 2507, it did not reach the level of the base metal. This reduction in corrosion resistance is a critical consideration for applications such as batteries. Therefore, although PWHT can enhance the corrosion resistance of SAF 2507, the residual differences compared with solution-heat-treated materials must be considered in practical applications.
(4) This study performed welding and PWHT on SDSS SAF 2507 for use as a material for Li-ion battery cases and analyzed resulting the corrosion resistance and alloy segregation. To use it for Li-ion batteries, plating technology to enhance electrical conductivity inside the case is necessary. However, research on this aspect has not been conducted, indicating a need for future studies on the plating behavior of welds.

## Figures and Tables

**Table 1:** Chemical composition of major alloys in SDSS SAF 2507, as determined by ICP-MS.

Element	C	N	Mn	Ni	Cr	Mo	Cu	Fe
Chemical composition	0.01	0.27	0.8	6.8	25.0	3.8	0.2	Bal.

**Table 2:** Chemical composition of austenite and ferrite after manufacturing processes (casting and solution annealing) of SDSS SAF 2507.

	Phase	Cr	Mo	Ni	N	Fe	PREN
Casting	Austenite	23.2 ± 2.1	3.1 ± 0.6	7.8 ± 1.4	0.49	Bal.	41.3
	Ferrite	26.7 ± 2.3	5.0 ± 0.8	5.8 ± 1.2	0.05	Bal.	43.9
Solution annealing	Austenite	23.4 ± 1.9	3.3 ± 0.7	7.9 ± 1.3	0.51	Bal.	42.5
	Ferrite	26.5 ± 1.8	4.6 ± 0.8	5.7 ± 1.2	0.05	Bal.	42.4

**Table 3:** Chemical composition with position (surface: 1–3, cross-section: 4–6) in Figure 4 of laser-welded super duplex stainless steel SAF 2507.

Site	Cr	Ni	Mo	N	O	Fe	PRE
1	24.7	6.5	3.7	0.27	3.3	Bal.	41.2
2	23.7	6.1	3.1	0.27	8.5	Bal.	38.3
3	23.1	5.8	3.0	0.27	16.1	Bal.	37.3
4	25.0	6.7	3.7	0.27	2.2	Bal.	41.5
5	24.4	6.4	3.5	0.27	6.5	Bal.	40.3
6	23.6	6.2	3.2	0.27	13.5	Bal.	38.5

**Table 4:** Chemical compositions of laser-welded SDSS SAF 2507 after post-weld heat treatment for 0–5 min.

Temperature	Phase	Cr	Mo	Ni	N	Fe	PREN
0 min	Austenite	25.0 ± 0.3	3.6 ± 0.1	12.5 ± 0.1	2.05	Bal.	69.7
	Ferrite	25.3 ± 0.4	3.8 ± 0.1	6.1 ± 0.2	0.05	Bal.	38.6
1 min	Austenite	24.4 ± 0.2	3.3 ± 0.1	7.4 ± 0.3	0.54	Bal.	43.9
	Ferrite	26.0 ± 0.4	4.1 ± 0.1	6.3 ± 0.2	0.05	Bal.	40.3
3 min	Austenite	24.1 ± 0.2	3.1 ± 0.1	7.2 ± 0.1	0.47	Bal.	41.9
	Ferrite	26.3 ± 0.3	4.5 ± 0.1	6.4 ± 0.2	0.05	Bal.	42.0
5 min	Austenite	24.1 ± 0.3	2.7 ± 0.1	7.2 ± 0.1	0.47	Bal.	40.5
	Ferrite	26.4 ± 0.3	4.8 ± 0.1	6.5 ± 0.2	0.05	Bal.	43.0

**Table 5:** OCP and potentiodynamic polarization curve results after post-weld heat treatment of laser-welded SDSS SAF 2507 in 3.5 wt% NaCl electrolyte solution.

PWHT Time	OCP Potential	Potentiodynamic Polarization Curve		
		E_corr_	I_corr_	E_pit_
0 min	−0.21 V	−0.16 V	2 × 10^−8^ A/cm^2^	1.00 V
1 min	−0.13 V	−0.10 V	8 × 10^−8^ A/cm^2^	1.02 V
3 min	0.03 V	0.05 V	1 × 10^−7^ A/cm^2^	1.11 V
5 min	−0.02 V	0.00 V	1 × 10^−7^ A/cm^2^	1.09 V

**Table 6:** Comparison of electrochemical properties with battery case materials of Al 1005, SAF 30400, and SDSS SAF 2507.

PWHT Time	OCP	E_pit_	CPT
Al 1005	−1.07 V	−0.65 V	Under 1 °C
SAF 30400	−0.10 V	0.20 V	Under 1 °C
SAF 2507 after PWHT	0.03 V	1.11 V	73 °C

## Data Availability

The original contributions presented in this study are included in the article. Further inquiries can be directed to the corresponding authors.
